# Molecular insights into symbiosis—mapping sterols in a marine flatworm-algae-system using high spatial resolution MALDI-2-MS imaging with ion mobility separation

**DOI:** 10.1007/s00216-020-03070-0

**Published:** 2020-12-03

**Authors:** Tanja Bien, Elizabeth A. Hambleton, Klaus Dreisewerd, Jens Soltwisch

**Affiliations:** 1 Institute of Hygiene, University of Münster, Robert-Koch-Str. 41, 48149 Münster, Germany; 2 Interdisciplinary Center for Clinical Research (IZKF), University of Münster, Domagkstr. 3, 48149 Münster, Germany; 3 Centre for Microbiology and Environmental Systems Science, Division of Microbial Ecology, University of Vienna, Althanstr. 14, 1090 Vienna, Austria

**Keywords:** MALDI, Sterols, Mass spectrometry imaging, MALDI-2, Waminoa acoel flatworm, Trapped ion mobility

## Abstract

*Waminoa* sp. acoel flatworms hosting *Symbiodiniaceae* and the related *Amphidinium* dinoflagellate algae are an interesting model system for symbiosis in marine environments. While the host provides a microhabitat and safety, the algae power the system by photosynthesis and supply the worm with nutrients. Among these nutrients are sterols, including cholesterol and numerous phytosterols. While it is widely accepted that these compounds are produced by the symbiotic dinoflagellates, their transfer to and fate within the sterol-auxotrophic *Waminoa* worm host as well as their role in its metabolism are unknown. Here we used matrix-assisted laser desorption ionization (MALDI) mass spectrometry imaging combined with laser-induced post-ionization and trapped ion mobility spectrometry (MALDI-2-TIMS-MSI) to map the spatial distribution of over 30 different sterol species in sections of the symbiotic system. The use of laser post-ionization crucially increased ion yields and allowed the recording of images with a pixel size of 5 μm. Trapped ion mobility spectrometry (TIMS) helped with the tentative assignment of over 30 sterol species. Correlation with anatomical features of the worm, revealed by host-derived phospholipid signals, and the location of the dinoflagellates, revealed by chlorophyll *a* signal, disclosed peculiar differences in the distribution of different sterol species (e.g. of cholesterol versus stigmasterol) within the receiving host. These findings point to sterol species-specific roles in the metabolism of *Waminoa* beyond a mere source of energy. They also underline the value of the MALDI-2-TIMS-MSI method to future research in the spatially resolved analysis of sterols.

**Supplementary Information:**

The online version contains supplementary material available at 10.1007/s00216-020-03070-0.

## Introduction

The photosynthetically powered transfer of nutrients from symbiotic algae to their hosts is a major driver of marine ecosystem function and evolution. The most widespread algal symbionts in animals, including the threatened reef-building corals, are the dinoflagellate *Symbiodiniaceae* [1, 2]. *Waminoa* sp. acoel flatworms (hereafter “*Waminoa*”), which host both *Symbiodiniaceae* and the related dinoflagellate *Amphidinium*, live epizoically on the surfaces of reef-building corals and compete with them for food and light [3–5]. Whereas the *Waminoa* symbiotic system is little described on the molecular level, investigations into *Symbiodiniaceae* symbiosis with cnidarians have shown lipid transfer to be important [6, 7]. Specifically, the symbiotic dinoflagellate algae synthesize a variety of sterols, including cholesterol, numerous phytosterols, and the gorgosterol uniquely produced by these symbiont species [8], which are transferred to the sterol-auxotrophic cnidarian hosts, as shown by gas chromatography (GC)-MS and mechanistic characterization of host-based importers [9, 10]. Many acoel flatworms are also sterol auxotrophs, some of which also receive sterols from their chlorophyte algae symbionts [11], and *Waminoa* is likewise sterol-auxotrophic. Yet it remains unknown whether sterol transfer occurs in these other *Symbiodiniaceae* symbioses with animal hosts besides the cnidarians. Further, the distribution of symbiont-produced sterols within host tissues and their function in host physiology have, to our knowledge, not been reported for any animal-algal symbiosis.

Mass spectrometry is arguably the most ubiquitously used technique in the analysis of sterols [12]. While historically GC-MS technique in combination with suitable derivatisation has been the analytical MS technique of choice [13–15], today electrospray ionization (ESI) MS [16] and especially matrix-assisted laser desorption/ionization (MALDI) [17–19] are increasingly used to analyze sterols [12]. However, because of poor ion yields, the analysis of neutral sterols like cholesterol or phytosterols is challenging with these techniques. Therefore, different derivatisation techniques have been introduced, based on Girards hydrazine reagents like Girard T reagent used to target keto groups [20–22], silylation [23], or pyridine sulfur trioxide [22], to significantly increase ion yields. Upon use of on-tissue derivatisation, these techniques have also been translated to the field of mass spectrometry imaging (MSI). This enabled the spatial analysis of cholesterol and its metabolites, vitamin D and its derivatives, and different steroids directly from tissue using desorption electrospray ionization (DESI), liquid extraction for surface analysis (LESA) and MALDI [22, 24, 25]. A problem, however, is the possible delocalization of analytes during the derivatization step. Moreover, LESA and DESI provide only a more limited spatial resolution in the tens of micrometer range, which is generally not sufficient to resolve features on a cellular scale.

A powerful method for the analysis of native neutral sterols at high spatial resolution and directly from tissue sections is secondary ion mass spectrometry (SIMS). Here [cholesterol-H_2_O+H]^+^ has been detected as prominent ion in the SIMS and NanoSIMS analysis of tissue and cell culture samples with a pixel size well below 1 μm [26–30].

However, because SIMS is prone to analyte fragmentation, discrimination of genuine molecular ions from fragments, originating, for instance, from cholesterol esters or chemical background, can be complicated. Modern hybrid SIMS instruments with high mass spectrometric resolution and the use of gas cluster ion beams (GCIB) (e.g., large argon or H_2_O clusters), or C_60_ beams, mitigate these problems, albeit these ion sources generally provide a somewhat lower spatial resolution [31, 32].

Detecting the same water-loss ion species as SIMS, MALDI-MS, and MSI of underivatized neutral sterols like cholesterol in positive ion mode has been reported with relatively poor sensitivity using different matrices [17–19, 33]. To improve the ion yields for sterols in a MALDI-MS(I) analysis, MALDI-2, a recently introduced laser post-ionization technique, can be employed. We and others have recently shown that in combination with state-of-the-art mass spectrometers, such as orbitrap and hybrid orthogonal-extracting time-of-flight (QTOF) mass spectrometers, MALDI-2 allows for the sensitive analysis of cholesterol, cholesterol esters, and vitamin D with high spatial and high mass spectrometric resolution [34, 35]. Compared to standard MALDI, up to 2–3 orders of magnitude higher ion signals were obtained upon use of the laser post-ionization module.

Additionally, different groups have demonstrated the use of silver nanoparticles as an inorganic matrix to visualize the distribution of [sterol+Ag]^+^ ions directly from tissue sections and from cell cultures [36–38]. In negative ion mode, neutral sterols have moreover been detected as sulfate species, using both nano-ESI and MALDI as ion sources [39, 40].

Chemically, all sterols are based on the same core structure and differ only in the number of carbons in the acyl chain, the state of oxidation, the number of carbon-carbon double bonds, and other alterations of the side groups. Consequently, the occurrence of isomeric ion species is very common in nature. While a differentiation of unknown isomers is not possible in an MS^1^ analysis alone, the use of standard substances and combination with preceding chromatographic separation as well as tandem MS can eventually enable structural elucidation [41]. To decipher full stereochemistry of purified substances without available standard, nuclear magnetic resonance (NMR) spectroscopy is therefore the technique of choice [42].

Introducing an additional layer of separation, the on-line combination of MALDI-MSI with ion mobility separation (IMS) is a powerful extension to the analysis of highly complex samples [43, 44]. The technique is particularly helpful in analyzing the additional chemical depth provided by MALDI-2. With IMS, ions are separated based on their collisional cross section in a bath gas prior to MS analysis. This allows for the individual analysis of ion species that are isobaric in the *m/z* domain [45]. Resulting three-dimensional data is usually presented in the form of mobilograms, where *m/z* information is commonly plotted on the abscissa, mobility separation is commonly plotted on the ordinate, and ion signal intensity is vizualized by a color scale. In the analysis of sterols, IMS has been used to aid quantitative studies of lipid extracts [10]. To our knowledge, IMS has, however, not been used for the separation of individual isomeric sterol species.

In this paper, we present a proof of concept study for the MALDI-2-TIMS-MSI analysis of neutral sterols from the symbiotic *Waminoa*/dinoflagellate system. This system is not only especially rich in sterol composition but also particularly small, with host tissue thickness averaging around 200 μm or less, and dimensions of single algae cells being about 9–13 μm in diameter. These boundary conditions necessitate both a particular sensitive MS analysis with high chemical depth and simultaneously a high spatial resolution with a pixel size of 5 μm. Next to an increased sensitivity gained by the use of MALDI-2, trapped ion mobility spectrometry (TIMS) is used to increase confidence in the assignment of sterol-derived ion signals on a MALDI-2 timsTOF flex instrument [45].

## Materials and methods

### Materials

Chloroform, methanol (MeOH), acetonitrile (ACN), and acetone were from Roth (Karlsruhe, Germany); 2,5-dihydroxyacetophenone (DHAP) was from Merck (Darmstadt, Germany); lathosterol and desmosterol were from Avanti Polar Lipids (Alabaster, AL, USA); and campesterol, cholesterol, and stigmasterol were from Sigma-Aldrich (Merck, Darmstadt, Germany). Embedding medium, prepared according to Nelson et al. [46], consisted of 5% 2-hydroxyethyl cellulose (*M*_v,avg_ ~ 90,000; Merck) and 10% gelatin (Merck) in ultrapure water.

### *Waminoa* culturing and cryosectioning

Worms were cultured in marine aquaria at 25 °C illuminated with two 60 cm 12 W/1020 lm LED SolarStinger SunStrips, with one each of “Marine” (15,000 K, 460 nm) and “DeepBlue” (400–460 nm) (Econlux, Cologne, Germany), under a light:dark regime of 12 h each. Aquaria were fed weekly with excess *Artemia* brine shrimp *nauplii* (exhibiting a sterol profile of nearly 100% cholesterol [10]). We note that, in addition to this source, dietary sterol input by the worm from grazing on coral polysaccharide mucus cannot be ruled out. Worms were isolated and starved at least 24 h before embedding. Animals were briefly relaxed in 0.5% MgCl_2_ in artificial seawater and then gently mixed in embedding medium, added to a cylindrical mold of a cut 2-mL microcentrifuge tube, and flash-frozen in liquid nitrogen. Samples were stored airtight at − 80 °C and equilibrated to − 20 °C prior to cryosectioning. Transverse sections of 14 μm thickness each were prepared using a Leica CM3050 S cryotome (− 20 °C chamber temperature, − 19 °C object temperature) and were thaw-mounted on SuperFrost glass slides (Thermo Scientific, Waltham, MA) or indium tin oxide (ITO)-coated glass slides (Merck, Darmstadt, Germany). After drying at room temperature (RT) for approx. 15 min, slides were sealed in individual conical tubes and frozen until further handling.

### Preparation of sterol standards

All standards were dissolved in chloroform to 2.5 mM. DHAP was prepared at 15 mg/mL in ACN:MeOH:H_2_O (8:1:1; v/v/v). For dried-droplet matrix preparations, standard and matrix solutions were mixed 1:1 by volume, followed by spotting of 1 μL on a stainless steel MALDI sample plate.

### Matrix application

Slides with worm sections were taken from the freezer and thawed and simultaneously dried at RT under a gentle stream of nitrogen. Matrix was applied directly after the sample appeared completely dry, in order to minimize time until start of the MSI analysis. For matrix application, a total amount of 30 mg DHAP, dissolved in 1.5 mL acetone, was transferred in the brass pan of a home-built sublimation chamber. The reservoir was preheated to 130 °C and, after solvent evaporation, the sample was placed on a water-cooled surface of ~ 4 °C directly over the pan with the sample pointing downward. The whole setup was then transferred to a vacuum of ~ 3 mbar and matrix was allowed to sublimate and redeposit for 6 min before the process was stopped by ventilation with N_2_. After removing the sample slide from the apparatus, it was immediately transferred to the ion source of the mass analyzer. The 6-min deposition resulted in a matrix coverage of 236 ± 34 μg/cm^2^ as derived from weight measurements of neat glass slides before and after sublimation under the same conditions.

### MALDI-2-MSI

MSI data were acquired on a modified timsTOF fleX instrument (Bruker Daltonik, Bremen, Germany) [45]. The mass resolving power of this hybrid QTOF-type instrument is about 40,000 (fwhm) in the investigated *m/z* range of 300–1000 and the mobility resolution is about 200. Primary material ejection and ionization were achieved with a SmartBeam 3D laser (actively Q-switched, frequency-tripled diode-pumped solid-state laser; wavelength: 355 nm) followed by laser post-ionization (actively Q-switched, frequency-quadrupled Nd:YAG, NL 204-1k-FH, EKSPLA, Vilnius, Lithuania; wavelength: 266 nm). The distance of the PI laser beam to the sample surface was set to about 500 μm and delay between the two lasers’ pulses, both operated at 1 kHz, was set to 10 μs. For material ejection, a scan range of 1 μm of the laser spot on the target was used, resulting in a round ablated area of 5 μm in diameter. The step size of the stage during the MSI run was set to 5 μm. Before each full MSI measurement, laser energies and number of shots per pixel were adjusted on parallel worm sections, mounted on the same sample slide, to obtain optimal ion signal intensities in the sterol range (*m/z* 300–500) of the spectrum. This step was necessary, because sample height may vary slightly when slides are mounted on the sample holder. For MALDI-2 measurements without additional TIMS separation, a mass range of *m/z* 300–1000 was used.

For measurements using TIMS, the *m/z* range was adjusted to 250–655 and 1/*k*_0_ was measured between 0.6 and 1.6 with a ramp time of 659 ms. Sterol standards were measured with the optimized (laser) conditions that were used for TIMS-on measurements. Spectra acquired during MSI were internally calibrated using a prominent diacylglycerolipid fragment signal ([DAG(34:1)]^+^) at *m/z* 577.5196, which was generally observed from tissue (see Fig. S1 of the Supplementary Information (ESM) for a presentation of the spatial distribution of this fragment in a worm section). The TIMS domain was calibrated using an ESI-L low molecular weight tune mix. While 1/*k*_0_ values undergo very little drift during individual imaging runs, the precision to determine absolute values in between measurements may suffer from small deviations in the ion source pressure and improper calibration. Therefore, no absolute 1/*k*_0_ values are compared between standard and tissue sections and all conclusions derived from TIMS are based on relative correlations, peak shapes, or separation of features within a single measurement. Shape and size of the areas to be measured with MSI were in all cases chosen to include the full section of the worm with at least 500 μm distance between the edges of the tissue and the borders of the image.

### Data handling

MALDI-2-MSI data were visualized with SCiLS Lab multivendor (MVS) software (Version 2020b Pro). For image visualization, an interval width of 15 ppm was used. MALDI-2-MS images are displayed after applying a weak denoising. MALDI-2-TIMS-MSI data were analyzed using TDF viewer software (Bruker Daltonik). Images were exported in a *.txt format using an *m/z* window of 0.005 and a 1/*k*_0_ window of 0.05–0.07. Flex-imaging software (Bruker Daltonik) was used for visualization without denoising.

Because largely different total ion counts were obtained from sampled areas with and without underlying tissue, all MSI images were generated without normalization. All raw data are available from the authors upon request.

### Tentative annotation of sterols

Because full structural information is not accessible with our method, the most probable isobaric sterol species connected to the respective sum formula are annotated, based on the available literature. For simplicity, these are denoted by their trivial names throughout the manuscript if available. In all other cases, chemical sum formulae are used to assign a species.

## Results and discussion

### Characteristics of sterol signals detected with MALDI-2-TIMS-MSI

As detected with regular MALDI without further derivatisation, and also with SIMS, in MALDI-2 sterols are, in the positive ion mode, predominantly detected as protonated species with the loss of water [sterol-H_2_O+H]^+^. In addition, most sterol species also produced radical ions of the form [sterol-H_2_O]^•+^, similarly observed in atmospheric pressure photoionization (APPI) [47]. Consequently, sterol signals are expected in the *m/z* region between about *m/z* 250 and 500 (see ESM Table S1 for a compilation of sterols and phytosterols commonly detected in marine systems including dinoflagellates). MALDI-2-MSI spectra generated directly from tissue in this *m/z* region are however generally highly complex with isobaric signals and overlapping peaks being more the rule than the exception. Matrix-derived signals mix with signals generated from tissue-derived metabolites, as well as possible fragments from larger analyte molecules (see ESM Fig. S2 for an exemplary mass spectrum). Consequently, even the relatively high resolving power of 40,000 and mass accuracy (≤ 5 ppm) of the employed mass spectrometer are not generally sufficient to assign less abundant sterol species reliably. We therefore included TIMS as an additional orthogonal separation technique into our MALDI-2-MSI workflow. Moving from the assignment of peaks in two-dimensional mass spectra to features in three-dimensional mobilograms de-convoluted signal superposition considerably. Mobility separation of isobaric compounds thereby significantly aids with tentative assignment of molecular identities (see ESM Fig. S3 for exemplary mobilograms) [45].

**Fig. 1:**
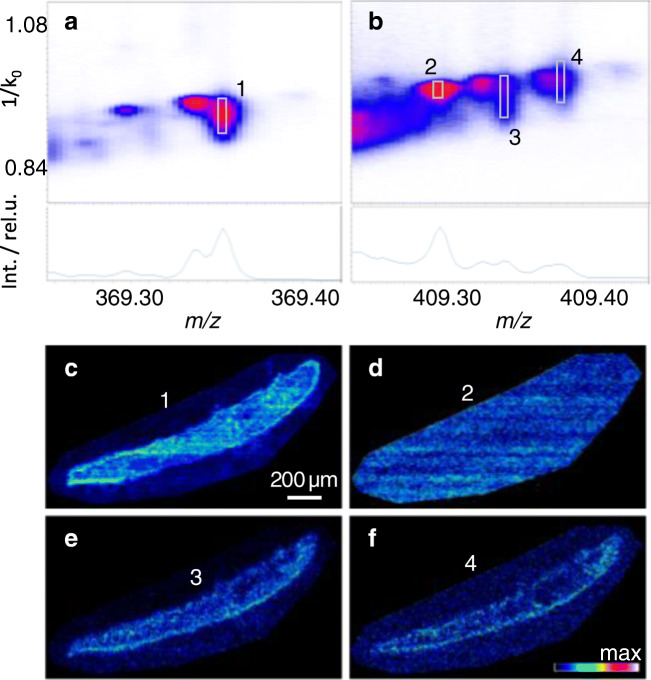
Mobilograms and mass spectra of cholesterol (**a**, feature 1), sterol C_29_H_46_O_2_ (**b**, 3), and gorgosterol (**b**, 4). Also shown is an exemplary feature assigned to matrix-derived chemical noise (**b**, 2); other matrix- as well as non-sterol-derived endogenous analyte signals showed similar compact features. Data were summed across a full section of *Waminoa *but also include areas of matrix-coated substrate next to the tissue. Signal intensity distribution vizualized for all four features (**c**–**f**)

**Fig. 2:**
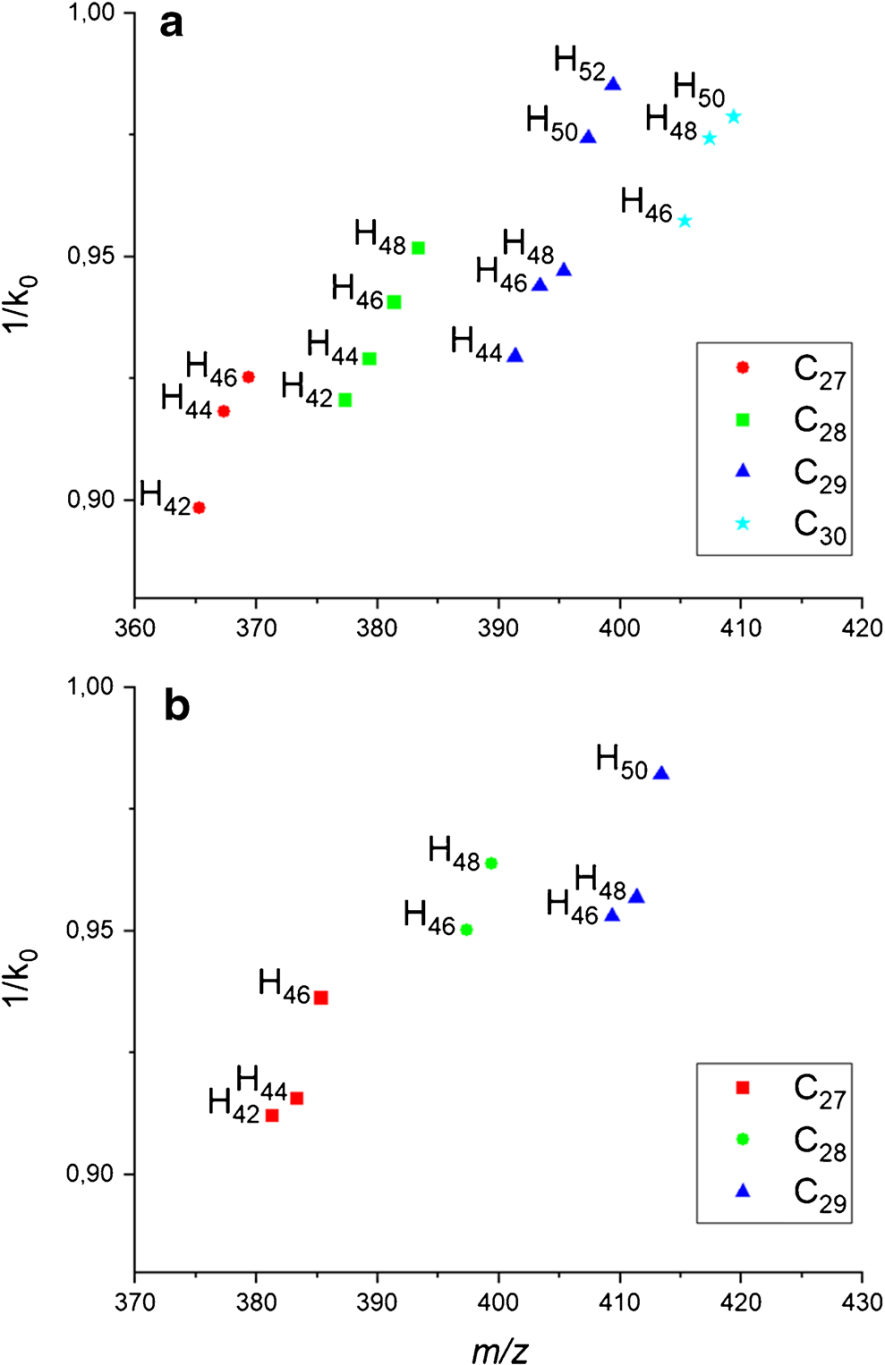
1/*k*_0_ values plotted against *m*/*z* for sterols containing (**a**) one and (**b**) two oxygens with different numbers of carbon atoms and degree of unsaturation. Data were derived from a MALDI-2-TIMS-MSI measurement summed over a full *Waminoa* section

**Fig. 3:**
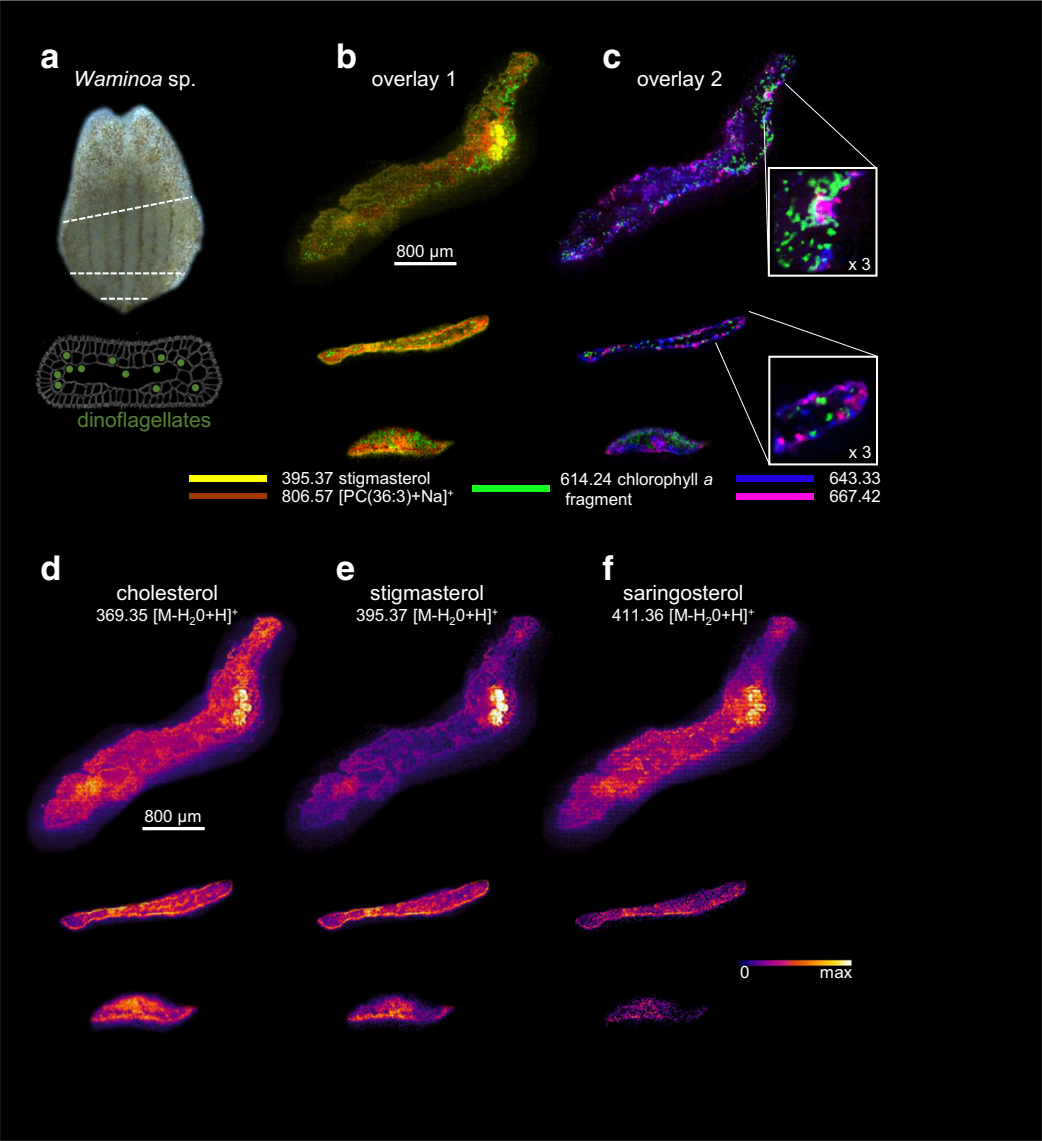
MALDI-2-MS imaging of *Waminoa* flatworms. Data were collected from sections of three individual flatworms and represent different “depth profiles.” **a** Snapshot of a *Waminoa* flatworm with approximate positions of the three sections produced for the MSI analyses, indicated by dotted lines, and schematic of a *Waminoa* cross section with symbiotic dinoflagellates. **b** Overlay of ion images of stigmasterol [M–H_2_O+H]^+^ (yellow) with [PC(36:3)+Na]^+^ (brown), representative of a group of phospholipids that are detected from animal tissue, and a chlorophyll *a* fragment, detected at *m/z* 614.24 (green), presentative of the two algae species. **c** Overlay ion images of the chlorophyll *a* fragment and two currently unknown species, detected at *m/z* values of 643.33 (blue) and 667.42 (pink), respectively. The two ions are representative of two groups of signals that are localized specifically within or close to dinoflagellates. MALDI-2-MS images of (tentative) cholesterol (**d**), stigmasterol (**e**), and saringosterol (**f**). MS images are presented with weak denoising, no normalization was applied. Figures denote measured *m/z* values of the registered ions. The scale bars plotted in b and d apply to all MS images in the figure

The analysis of sterol standards (e.g., cholesterol and stigmasterol) revealed that no sizeable in-source fragmentation other than the loss of water is generated with MALDI-2. Using TIMS, these sterol ion species produce characteristic features in a mobilogram (ESM Fig. S4). While their peak shape in the *m/z* dimension is normal, the peak shape in the mobility domain is peculiar. Compared to phospholipid or matrix-derived ions, peak shapes are considerably broadened for all tested sterols. This peculiarity limits the ability to separate sterols in the mobility domain and hampers the identification of isomeric sterol structures (see ESM Fig. S4 for mobilograms of two isobaric standards). Beneficially, however, it leads to a characteristic feature shape that appears exclusive to sterols in the mobilogram as demonstrated in Fig. 1. While cholesterol (Fig. 1a, 1), C_29_H_46_O_2_ (Fig. 1b, 3), and gorgosterol (Fig. 1b, 4) display a feature “drawn-out” in the mobility dimension, the feature tentatively assigned to chemical noise in Fig. 1b, 2, is much more compact. Similarly, compact features were also detected for non-sterol lipids such as phospholipids in the respective mass range.

Mechanistically, it may be speculated that the initial attachment of charge to the 3β-hydroxyl group [48] and the subsequent loss of water forming the detected ion species allow for the generation of isomeric structures in the gas phase. The ionic form possibly enables charge rearrangement processes that lead to the opening of one or more rings in something like a retro-cyclization reaction (see scheme in ESM Fig. S5). Consequently, an unknown number of tautomers with distinctively different collisional cross sections may contribute to the ion mobility peak broadening.

### Tentative assignment of sterol species detected from the symbiotic system

Tentative assignment of sterol signals was performed in a multi-step process. As a starting point, we compiled a list of sterols that are commonly detected in marine systems with 123 entries (ESM Table S-1). To identify features in the mobilogram connected to [sterol-H_2_O+H]^+^ ion species from this list, we allowed for a deviation of 5 ppm from the theoretical values in the *m/z* dimension of the MSI data. Features meeting this criterion were classified with values ranging from 0 to 4, according to their characteristic, indicatively broad peak shape in the mobility domain and overlap with (near-) isobaric features. In the grading system, 0 stands for no trace, and 4 for a clear and differentiated feature (see ESM Fig. S6, for examples). For features graded 1–4, images were extracted using an *m/z* window of 0.005 and a 1/*k*_0_ window according to the peak width (0.05–0.07). Resulting images were again graded on a scale from 0 to 4, based on the image clarity and contrast. Zero describes no visible distribution in the image. Grades from 1 to 4 differentiate between the shape of the worm barely discernable from background noise (grade 1) and a clear distribution with high signal-to-noise (grade 4), as demonstrated in Fig. S7 (see ESM) with exemplary ion images. Grades for feature and image clarity were multiplied and only features with a resulting combined grade ≥ 3 were considered for further analysis. Table 1 shows the resulting number of 32 tentatively assigned sterols with their grading and chemical sum formula.

**Table 1:** List of sterols detected by MALDI-2-TIMS-MSI in the *Waminoa*/dinoflagellate system using a classification system based on the individual image distribution quality and feature clarity; all tentative structural assignments based on accurate mass (5 ppm) and the cited literature; data were extracted from a single MALDI-2-TIMS-MSI measurement of a section of a *Waminoa*

Chemical composition			[M-H_2_O+H]^+^ (calc)	[M-H_2_O+H]^+^ (exp.)	Mass error (ppm)*	1/*k*_0_	Image distribution	Feature clarity	Combined grade	Sterol ID^#^	Ref
C	H	O									
26	42	1	353.321	353.320	3.8	0.91	1	4	4	24-nor-cholest-5,22E-dien-3β-ol	[49]
27	42	1	365.321	365.321	1.0	0.90	2	3	6	glaucasterol, cholest-5-en-23-yn-3β-ol,	[49]
27	44	1	367.336	367.337	0.9	0.92	4	3	12	desmosterol	[49]
27	46	1	369.352	369.353	2.9	0.93	4	4	16	cholesterol	[10]
28	42	1	377.321	377.319	4.8	0.92	4	4	16	dehydroergosterol	^‡^
28	44	1	379.336	379.336	1.0	0.93	2	3	6	ergosterol	[49]
28	46	1	381.352	381.352	1.4	0.94	3	3	9	brassicasterol	[50]
28	48	1	383.368	383.367	1.2	0.95	2	3	6	campesterol	[10]
29	44	1	391.336	391.335	4.5	0.93	3	3	9	conicasterol B	[51]
29	46	1	393.352	393.352	0.4	0.94	4	4	16	calysterol	[49]
29	48	1	395.368	395.369	3.4	0.95	4	4	16	stigmasterol	[10]
29	50	1	397.383	397.382	3.8	0.97	3	2	6	β-sitosterol	[9]
29	52	1	399.399	399.398	3.7	0.99	1	4	4	stigmastanol	[10]
30	46	1	405.352	405.351	2.0	0.96	3	2	6	theonellasterol B	[51]
30	48	1	407.368	407.367	2.4	0.97	3	2	6	nervisterol	[52]
30	50	1	409.383	409.382	3.7	0.98	3	2	6	gorgosterol	[49]
32	54	1	437.415	437.413	4.6	1.02	3	2	6	24-(1-ethyl-2-methyl-2-propenyl)-27-norcholest-7-en-3-ol	[49]
21	28	2	295.206	295.206	0.1	0.79	2	4	8	18-hydroxypregna-1,4,20-trien-3-one	[49]
23	38	2	329.284	329.286	3.5	0.87	1	4	4		^‡^
24	42	2	345.316	345.315	3.0	0.89	2	4	8		^‡^
27	42	2	381.316	381.317	2.5	0.91	4	3	12	chabrosterol	[51]
27	44	2	383.331	383.332	1.6	0.92	4	3	12	24-ketocholesterol	[49]
27	46	2	385.347	385.348	1.2	0.94	4	4	16	24-ketocholestanol	[49]
28	46	2	397.347	397.348	1.9	0.95	4	2	8	22,23-epoxycampesterol	[49]
28	48	2	399.363	399.362	0.7	0.96	3	2	6	22,23-epoxy-5β-campestan-3β-ol	[49]
29	46	2	409.347	409.347	0.6	0.95	4	3	12	22S,23S-epoxy-5α-stigmast-8(9),14(15)-dien-3β-ol	[49]
29	48	2	411.363	411.364	2.2	0.96	4	3	12	saringosterol	[49]
29	50	2	413.378	413.378	0.3	0.98	3	3	9	22S-hydroxysitosterol	[53]
30	50	2	425.378	425.379	2.0	0.98	4	1	4		^‡^
21	32	3	315.232	315.233	2.9	0.82	3	2	6	3β,6α-dihydroxy-5α-pregn-9(11)-en-20-one	[49]
23	38	3	345.279	345.280	1.9	0.88	3	2	6		^‡^
29	50	3	429.373	429.375	3.4	0.99	4	4	16	nebrosteroid M	[51]

*With internal calibration; ^#^tentative assignment of isomeric species based on literature; ^‡^identified in *Symbiodiniaceae*-containing marine systems based on unpublished results

Next to using the characteristic feature shape in the mobility domain to identify features connected to sterols, TIMS allows to extract 1/*k*_0_ value indicative of the collisional cross section of the observed ion species. Sterols with slight changes to their molecular structure can be expected to show systematic changes in their ion mobility based on the number of carbons and hydrogens added. Figure 2 displays mobility values plotted against measured *m/z* values for sterols containing one (Fig. 2a) and two (Fig. 2b) oxygens in their unperturbed structure. Groups of sterols containing the same number of carbons can clearly be discerned. Collisional cross sections within these groups strongly decrease with the degree of unsaturation inside the molecule. As a main result, all sterols detected from the animal/plant system fall into a coherent pattern. Making use of this consistent correlation within the chemical context of the core structure, it should be possible to identify isobaric outliers and exclude them from further analysis; in our sample system, none was however identified. The smaller number of detected sterol species containing two oxygens (Fig. 2b) leads to a smaller “chemical context” and renders correlation in the graph less conclusive. For sterol species containing three oxygens, the “chemical context” is not sufficient to draw any conclusions with regard to similarities in core structure.

### MS imaging of sterols

Because of the small size of *Waminoa*, with typical maximum dimensions of 4 mm length, 2 mm width, and 200 μm thickness, sections were measured with the smallest available pixel size of 5 μm. As the dinoflagellate cells have diameters of approx. 9–13 μm and reside predominantly inside host cells (Fig. 3a), this enables to resolve the algae and their direct surroundings on a molecular level. Moreover, the ion images of some phospholipid species can be used to outline the morphology (i.e., tissue compartments) of the flatworm. The resulting images reveal signal intensity distributions measured with MALDI-2-TIMS-MSI for sterol species containing one, two, and three oxygens (see ESM Fig. S8). C27 sterols are found throughout the whole worm. Among this group, cholesterol (C_27_H_46_O) produces the highest ion intensity. With increasing number of carbons, sterol species (e.g., gorgosterol (C_30_H_50_O)) are more localized to the edge of the animal (Fig. 1f), with C_29_H_46_O and C_29_H_48_O as well as C_29_H_48_O_2_, presumably calysterol, stigmasterol, and saringosterol, forming an exception to this rule. Some species containing three oxygens are more closely co-localized with the algae, and nebrosteroid M (C_29_H_50_O_3_) is the only tentatively assigned sterol predominantly detected directly from the dinoflagellates.

While being exceedingly helpful for feature assignment, the employment of the TIMS modality slows data acquisition speed to about 2 pixels/s and somewhat limits the accessible *m/z* range. However, building on information from measurements with TIMS, features with high grades for their feature clarity that show little to no overlap in the *m/z* domain can also be analyzed using MALDI-2-MSI without mobility separation, enabling the analysis of larger scan areas as well as a broader mass range in a reasonable time. Notably, a number of sterol species highly graded in MALDI-2-TIMS-MSI display a poor signal-to-noise in MALDI-2-MSI or are not detected at all (e.g., nebrosteroid M) when no TIMS is used.

Figure 3d–f shows the distributions of cholesterol (C_27_H_46_O), stigmasterol (C_29_H_48_O), and saringosterol (C_29_H_48_O_2_) in three sections of three different flatworms recorded using MALDI-2-MSI. The approximate positions of the sections and a schematic of the dinoflagellate symbionts within the animal are shown in Fig. 3a. While cholesterol is distributed almost homogenously throughout the tissue, the cross section through the center of one of the animals (top) reveals a higher concentration of stigmasterol within an area corresponding to the syncytial gut, possibly containing gonads. Saringosterol is distributed similar to cholesterol in the center of the animal, but shows depleted signal intensity in the outer parts.

Reflecting artefacts from sample preparation, sterols are often also detected outside of the tissue in the surrounding embedding medium and with signal intensities generally decreasing with distance. Next to smearing during sample preparation, we observed a temperature and time dependence of the effect, as it appears strongest when the period of time between preparation and measurement exceeds a few hours to days while the sample is not sufficiently cooled. In these samples, cholesterol and other sterol species can be detected up to 200 μm away from the tissue (ESM Fig. S9). A similarly strong diffusion of cholesterol was also reported previously in an MS imaging context [54]. Additionally, the inner part of the animals is more prone to these smearing effects as compared to smaller sections from the outer edge, pointing to differences in the distribution and embedding of cholesterol. Consequently, MS images of sterols with high lateral resolution have to be interpreted with due caution.

Apart from the variety of sterols, numerous signals are identified in the MSI data that correlate well with the structure of the worms. Based on accurate mass, a number of these signals can tentatively be assigned as phospholipids (see Fig. S2 for an example mass spectrum). Representative for this group, the distribution of [PC(36:3)+Na]^+^, where PC stands for phosphatidylcholine, is displayed in Fig. 3b (brown) (For more examples of phospholipid images, refer to ESM Fig. S1). Other signal intensity distributions correlate with the dinoflagellates. Interestingly, three groups of spatial features, putatively assigned to the single-cellular algae by their shape and size, can clearly be discerned based on these signals (Fig. 3c, insets). The most intense of these signals can be assigned to the photosynthetic molecule chlorophyll *a*, which appears to undergo in-source fragmentation. In support, tandem MS of the precursor ion at *m/z* 892 produces the same fragment ion species (ESM Fig. S10). However, the molecular identities of the ion species associated with the other two groups, as well as their assignment to possible sub-populations of one or both symbiotic dinoflagellate species (or perhaps even as-yet-unidentified host features), remain unknown.

Interestingly, although it is well known that these worms receive their phytosterols from the dinoflagellates and do not produce them themselves, no correlation between the algae signals and most sterol signals can be observed. This could be due to relatively fast absorption of algae-produced sterols and their distribution within the organism, as well as a possible slow turnover of sterols in host tissues such that concentrations in host membranes are sufficiently high as to appear indistinguishable from the algal sources. Finally, it is possible that some sterols could be modified or converted by host enzymes [55], which warrants future investigation.

## Conclusion

The combination of MALDI-2 and TIMS enables the sensitive analysis of a large number of sterol species from tissue without prior derivatisation. The boosted signal intensity enabled by laser post-ionization allowed us to perform MS imaging experiments with a pixel size as low as 5 μm, approaching a cellular resolution for algal systems. While MALDI-2 critically increases signal intensity, TIMS crucially aids with the tentative assignment of sterols. In addition to accurate mass measurement within 5 ppm, the characteristic shape of the sterol features in the mobilogram as well as a proper placement of 1/*k*_0_ values within the chemical context of the investigated sterol species help to increase the confidence of the assignment significantly. Moreover, careful feature selection in the 3-dimensional domain of the mobilogram effectively increases signal-to-noise ratios. All sterol-derived [M-H_2_O+H]^+^ ions display a peculiar peak broadening in the 1/*k*_0_ domain. Consequently, MALDI-2-TIMS-MS analysis is not able to differentiate between isomeric ion species, despite expected differences in collisional cross sections between the intact molecules.

Besides the targeted analysis of sterols, collected MALDI-2-MSI data contains spatial information about a plethora of other molecular ion species. Next to different phospholipid species co-located with regions of the *Waminoa* host, signals were detected that are exclusively co-located with the symbiotic dinoflagellates, including intact chlorophyll *a* and some of its fragments as well as yet unidentified species. Although beyond the scope of this proof of concept study, in principle this would allow for an in-depth analysis of a broad spectrum of molecules located within host and symbionts.

Overall, MALDI-2-TIMS-MSI enabled the detection and localization of 32 individual sterol species. The observed distribution of symbiont-produced sterols within host flatworm tissues reveals that sterol transfer is a conserved element of *Symbiodiniaceae* symbioses in host backgrounds across phyla, from cnidarians [9, 10] to acoel flatworms. Surprisingly, the different spatial distribution of stigmasterol and to a lesser extent calysterol and saringosterol, compared to cholesterol in an area corresponding to the syncytial gut and possibly gonads, could indicate an unprecedented differential use of the various symbiont-produced sterols. The accumulation of these sterols could be based on selectively higher transport rates to this region, or to a particularly prolonged residence time before metabolization, or a combination of these and other factors, yet the mechanisms and function remain unknown. Among the variety of closely related symbiont-produced sterols on offer, what drives the concentration of stigmasterol in particular? What role does stigmasterol play in host physiology? How do the two algal symbiont types relatively contribute to the production of stigmasterol as well as other sterols and lipids? In the future, such questions can be addressed with the methods established here, which allow for complex sterol imaging in small, dynamic systems such as ecologically important symbioses as well as various other intricate tissue environments.

## Supplementary information

ESM 1 (PDF 2.12 MB)
