# Supplementary material for: Molecular insights into symbiosis—mapping sterols in a marine flatworm-algae-system using high spatial resolution MALDI-2-MS imaging with ion mobility separation
Source: Anal Bioanal Chem. 2020 Dec 3;413(10):2767–77. doi: 10.1007/s00216-020-03070-0 (PMC8007520; doi:10.1007/s00216-020-03070-0)
Supplement: Supplementary file 1 — (PDF 2.12 MB) [file 216_2020_3070_MOESM1_ESM.pdf]

Supplementary Information

For

Molecular insights into symbiosis—mapping sterols in a marine flatworm-algae-system using high spatial resolution MALDI-2-MS imaging with ion mobility separation

Tanja Bien[1,2*], Elizabeth A. Hambleton[3*], Klaus Dreisewerd[1,2], Jens Soltwisch[1,2,#]

[1]Institute of Hygiene, University of Münster, Robert-Koch-Str. 41, 48149 Münster, Germany

[2]Interdisciplinary Center for Clinical Research (IZKF), University of Münster, Domagkstr. 3, 48149 Münster, Germany

[3]Centre for Microbiology and Environmental Systems Science, Division of Microbial Ecology, University of Vienna, Althanstr. 14, 1090 Vienna, Austria

* Contributed equally to this work

# Correspondence addressed to jenssol@uni-muenster.de

ORCID:

Elizabeth A. Hambleton: https://orcid.org/0000-0003-0204-5037

Klaus Dreisewerd: https://orcid.org/0000-0002-7619-808X

Jens Soltwisch: https://orcid.org/0000-0002-0258-1561

Table of Contents

Table S1 pg. S2 – S5

Figure S1 – S10 pg. S6 – S13

Supplementary References pg. S13 – S 14

Table S1 List of sterols commonly detected in marine systems as described in the referenced literature or identified in *Symbiodiniaceae*-containing marine systems based on unpublished results

Sterol ID	**Formula**			***M***	***[M-H_2O+H]$^+$***	**References**
	C	***H***	***O***			
**estrone**	18	22	2	270.16	253.16	
**β-estradiol**	18	24	2	272.18	255.17	
**estradiol-17α**	18	24	2	272.18	255.17	
**nandrolone**	18	26	2	274.19	257.19	
**19-norandrosterone**	18	28	2	276.21	259.21	
**19-nor-5-androstenediol**	18	28	2	276.21	259.21	
**estrane-3α,17α-diol**	18	30	2	278.22	261.22	
	18	20	3	284.14	267.14	
	18	22	3	286.16	269.15	
**dehydrotestosterone**	19	26	2	286.19	269.19	(Armoza-Zvuloni et al., 2014)
**pregna-5-20-dien-3β-ol**	20	30	1	286.23	269.23	(Kerr & Baker, 1991)
	18	24	3	288.17	271.17	
**testosterone**	19	28	2	288.21	271.21	(Armoza-Zvuloni et al., 2014)
**5β-dihydrotestosterone**	19	30	2	290.22	273.22	
**androstenediol**	19	30	2	290.22	273.22	
**3β-androstanediol**	19	32	2	292.24	275.24	
	19	24	3	300.17	283.17	
	19	24	3	300.17	283.17	
**methandrostenolone**	20	28	2	300.21	283.21	
	19	26	3	302.19	285.19	
	19	26	3	302.19	285.19	
**17-methyltestosterone**	20	30	2	302.22	285.22	
	19	28	3	304.20	287.20	
	19	28	3	304.20	287.20	
**mestanolone**	20	32	2	304.24	287.24	
**methandriol**	20	32	2	304.24	287.24	
	19	30	3	306.22	289.22	
	19	30	3	306.22	289.22	
**18-hydroxypregna-1,4,20-trien-3-**	21	28	2	312.21	295.21	(Kerr & Baker, 1991)
**16,17-didehydropregnenolone**	21	30	2	314.22	297.22	
**pregnenolone**	21	32	2	316.24	299.24	

**allopregnanolone**	21	34	2	318.26	301.25	
**3α,20β-pregnanediol**	21	36	2	320.27	303.27	
	21	30	3	330.22	313.22	
	21	32	3	332.24	315.23	
	21	32	3	332.24	315.23	
	21	34	3	334.25	317.25	
	21	34	3	334.25	317.25	
	22	32	3	344.24	327.23	
**3β-hydroxy-24-norchol-5-en-23-al**	23	36	2	344.27	327.27	(Kerr & Baker, 1991)
**24-nor-5β-chol-22-ene-3α,12α-diol**	23	38	2	346.29	329.28	
**11β,21-dihydroxy-5β-pregnane-**	21	32	4	348.23	331.23	
**3β,15β,17α-trihydroxy-**	21	32	4	348.23	331.23	
**tetrahydrocorticosterone**	21	34	4	350.25	333.24	
**26,27-dinor-cholest-5-en-23-yn-**	25	38	1	354.29	337.29	(Kerr & Baker, 1991)
**26,27-bisnor-22-dehydro-**	25	40	1	356.31	339.31	(Kerr & Baker, 1991)
**cortisone**	21	28	5	360.19	343.19	
**5-dihydrocortisone**	21	30	5	362.21	345.21	
	23	38	3	362.28	345.28	
**5β-cholane-3α,24-diol**	24	42	2	362.32	345.32	
	23	40	3	364.30	347.29	
**24-nor-cholest-5,22E-dien-3β-ol**	26	42	1	370.32	353.32	(Kerr & Baker, 1991)
**24-norcholesterol**	26	44	1	372.34	355.34	(Kerr & Baker, 1991)
**19-norcholestanol**	26	46	1	374.35	357.35	(Kerr & Baker, 1991)
	24	42	3	378.31	361.31	(Kerr & Baker, 1991)
**12β-hydroxy-24-norcholesta-**	26	38	2	382.29	365.28	(Kerr & Baker, 1991)
**glaucasterol**	27	42	1	382.32	365.32	(Kerr & Baker, 1991)
**desmosterol**	27	44	1	384.34	367.34	(Kerr & Baker, 1991)
**cholesterol**	27	46	1	386.35	369.35	
**dihydrocholesterol**	27	48	1	388.37	371.37	
**dehydroergosterol**	28	42	1	394.32	377.32	
**ergosterol**	28	44	1	396.34	379.34	(Kerr & Baker, 1991)
**chabrosterol**	27	42	2	398.32	381.32	(Blunt et al., 2013)
**brassicasterol**	28	46	1	398.35	381.35	(Sjövall & Danielsson, 1985)
	26	40	3	400.30	383.29	
**24,25-epoxy-cholesterol**	27	44	2	400.33	383.33	(Kerr & Baker, 1991)

**(22,23-dinor)-24-vinyl-cholest-5-**	27	44	2	400.33	383.33	
**campesterol**	28	48	1	400.37	383.37	
	26	42	3	402.31	385.31	
**24-ketocholestanol**	27	46	2	402.35	385.35	(Kerr & Baker, 1991)
**25-hydroxy-cholesterol**	27	46	2	402.35	385.35	
**campestanol**	28	50	1	402.39	385.38	
**6α-hydroxycholestanol**	27	48	2	404.37	387.36	
**conicasterol B**	29	44	1	408.34	391.34	(Blunt et al., 2013)
**calysterol**	29	46	1	410.35	393.35	(Kerr & Baker, 1991)
	27	40	3	412.30	395.29	
**stigmasterol**	29	48	1	412.37	395.37	
	27	42	3	414.31	397.31	
	27	42	3	414.31	397.31	
**22,23-epoxycampesterol**	28	46	2	414.35	397.35	(Kerr & Baker, 1991)
**24-methylene-cholest-5-en-3β,19-**	28	46	2	414.35	397.35	(Kerr & Baker, 1991)
**β-sitosterol**	29	50	1	414.39	397.38	(Revel et al., 2016)
	27	44	3	416.33	399.33	
	27	44	3	416.33	399.33	
	27	44	3	416.33	399.33	
**22,23-epoxy-5β-campestan-3β-ol**	28	48	2	416.37	399.36	(Kerr & Baker, 1991)
**22S-hydroxycampesterol**	28	48	2	416.37	399.36	(Ohnishi et al., 2009)
**stigmastanol**	29	52	1	416.40	399.40	
**20,22-dihydroxycholesterol**	27	46	3	418.34	401.34	
**6-deoxocathasterone**	28	50	2	418.38	401.38	(Ohnishi et al., 2009)
**hipposterol**	27	48	3	420.36	403.36	(Kerr & Baker, 1991)
**theonellasterol B**	30	46	1	422.35	405.35	(Blunt et al., 2013)
**nervisterol**	30	48	1	424.37	407.37	(Calderón et al., 2004)
**stoloniferone A**	28	42	3	426.31	409.31	(Kerr & Baker, 1991)
**stoloniferone O**	28	42	3	426.31	409.31	(Blunt et al., 2013)
**24-vinyloxy-cholest-5,23Z-dien-3β-**	29	46	2	426.35	409.35	(Kerr & Baker, 1991)
**gorgosterol**	30	50	1	426.39	409.38	(Kerr & Baker, 1991)
**nebrosteroid J**	28	44	3	428.33	411.33	(Blunt et al., 2013)
**stoloniferone E**	28	44	3	428.33	411.33	(Blunt et al., 2013)
**(25R)-5α,6α-epoxy-24R,26R-**	29	48	2	428.37	411.36	(Blunt et al., 2013)
**saringosterol**	29	48	2	428.37	411.36	(Kerr & Baker, 1991)

**gorgostanol**	30	52	1	428.40	411.40	(Kerr & Baker, 1991)
**nebrosteroid K**	28	46	3	430.34	413.34	(Blunt et al., 2013)
**5α,6β-dihydroxy-24-**	28	46	3	430.34	413.34	(Kerr & Baker, 1991)
**nephalsterol B**	28	46	3	430.34	413.34	(Kerr & Baker, 1991)
**22S-hydroxysitosterol**	29	50	2	430.38	413.38	(Ohnishi et al., 2009)
**4-methylstigmastan-3-ol**	30	54	1	430.42	413.41	
**cathasterone**	28	48	3	432.36	415.36	
**22,23-dihydroxycampesterol**	28	48	3	432.36	415.36	(Blunt et al., 2013)
**6-deoxotyphasterol**	28	50	3	434.38	417.37	
**ophirasterol**	31	50	1	438.39	421.38	(Calderón et al., 2004)
**stoloniferone D**	29	44	3	440.33	423.33	(Kerr & Baker, 1991)
**theonellasterol C**	30	48	2	440.37	423.36	(Blunt et al., 2013)
**sutinasterol**	31	52	1	440.40	423.40	(Kerr & Baker, 1991)
**nebrosteroid I**	29	46	3	442.34	425.34	(Blunt et al., 2013)
**3β-hydroxy-lanost-8-en-30-al**	30	50	2	442.38	425.38	
**32-hydroxylanosterol**	30	50	2	442.38	425.38	
**conicasterol D**	29	48	3	444.36	427.36	(Blunt et al., 2013)
**saoussazine**	30	52	2	444.40	427.39	(Blunt et al., 2013)
**nebrosteroid M**	29	50	3	446.38	429.37	(Blunt et al., 2013)
**(3β,5α,6β)-stigmastane-3,5,6-triol**	29	52	3	448.39	431.39	
**24-(1-ethyl-2-methyl-2-propenyl)-**	32	54	1	454.42	437.41	(Kerr & Baker, 1991)
**conicasterol C**	30	50	3	458.38	441.37	(Blunt et al., 2013)
**theonellasterol D**	31	52	3	472.39	455.39	(Blunt et al., 2013)

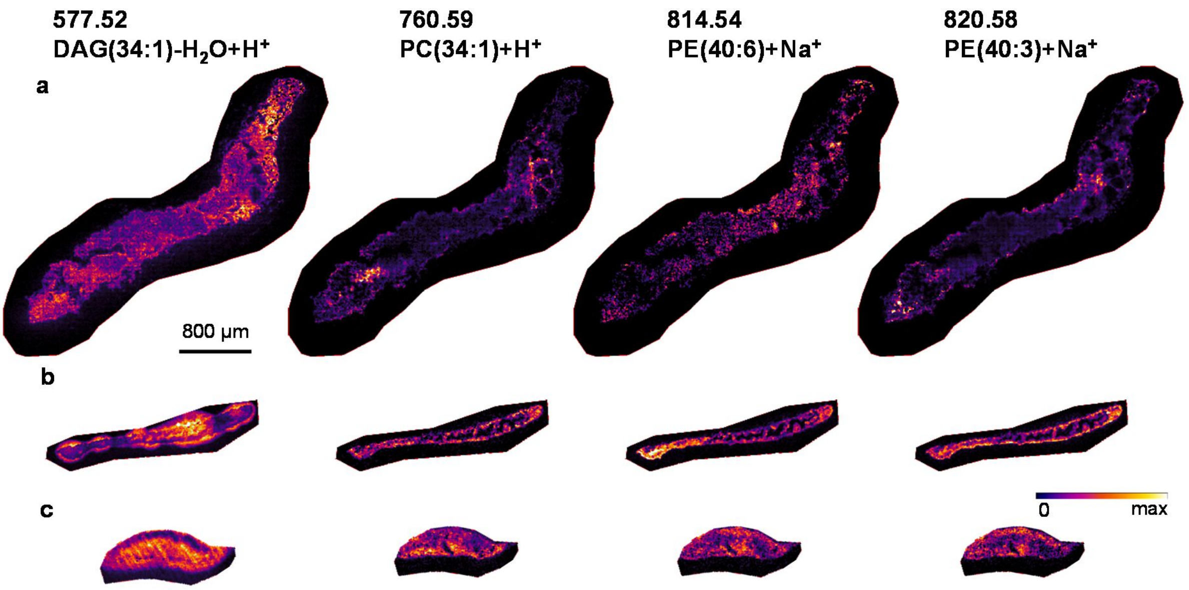

Fig. S1 Ion signal intensity distributions of different phospholipids or phospholipid fragments in three sections of individual flatworms (**a**-**c**) representing different depth-profiles. The scale bar is applicable to all sub figures

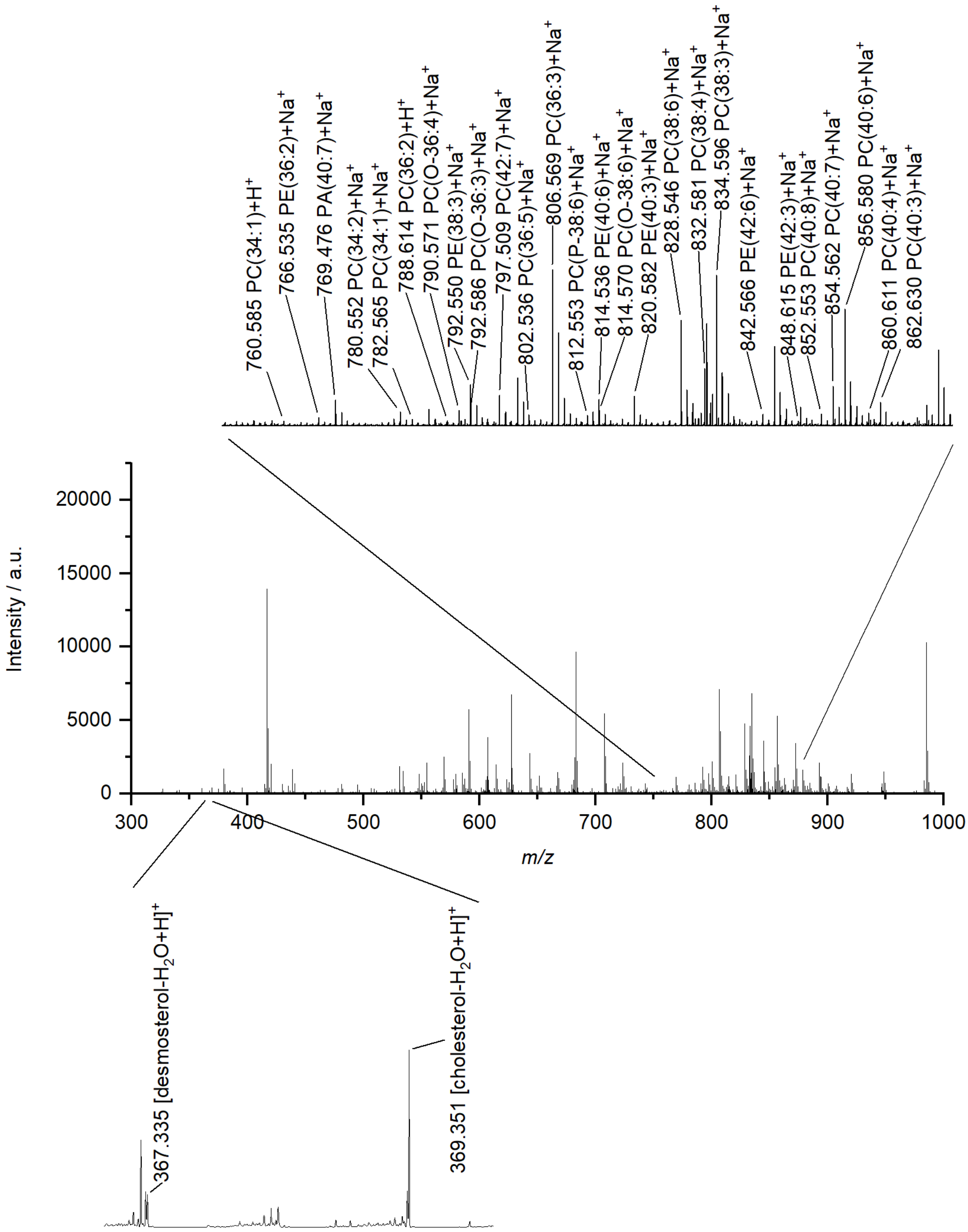

Fig. S2 Full mass spectrum of a full MALDI-2-MSI experiment on a section of *waminoa* sp. (middle) and cut-outs representing the phospholipid region (top) and a 2.5 Da wide window including two sterol ion species (bottom). In the top cut-out PC and PE species were tentatively assigned based on accurate mass.

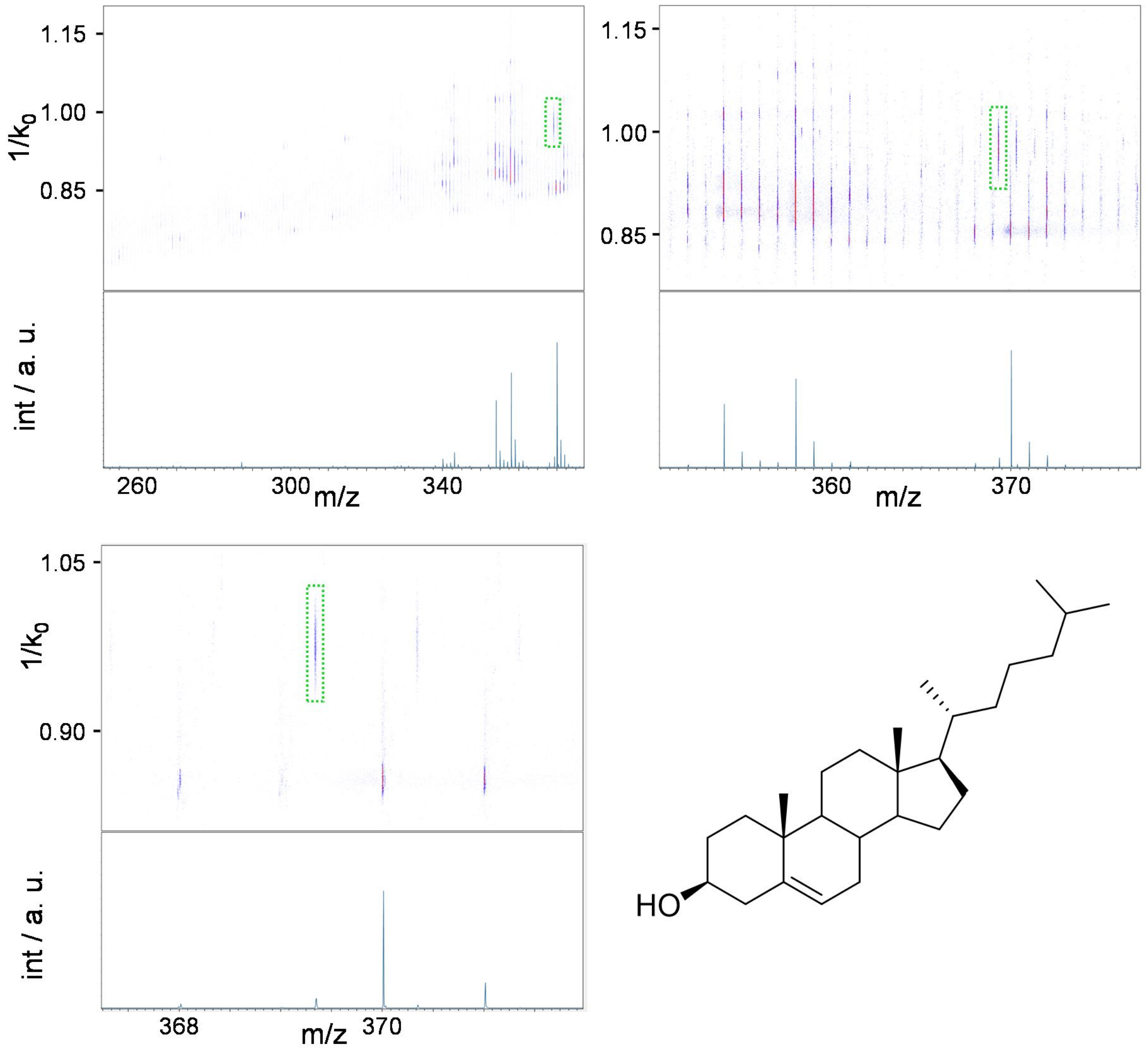

Fig. S3 Mass spectrum and mobilogram of a full MALDI-2-TIMS-MSI experiment on a section of *waminoa* sp. zooming in on cholesterol and the molecular structure of cholesterol.

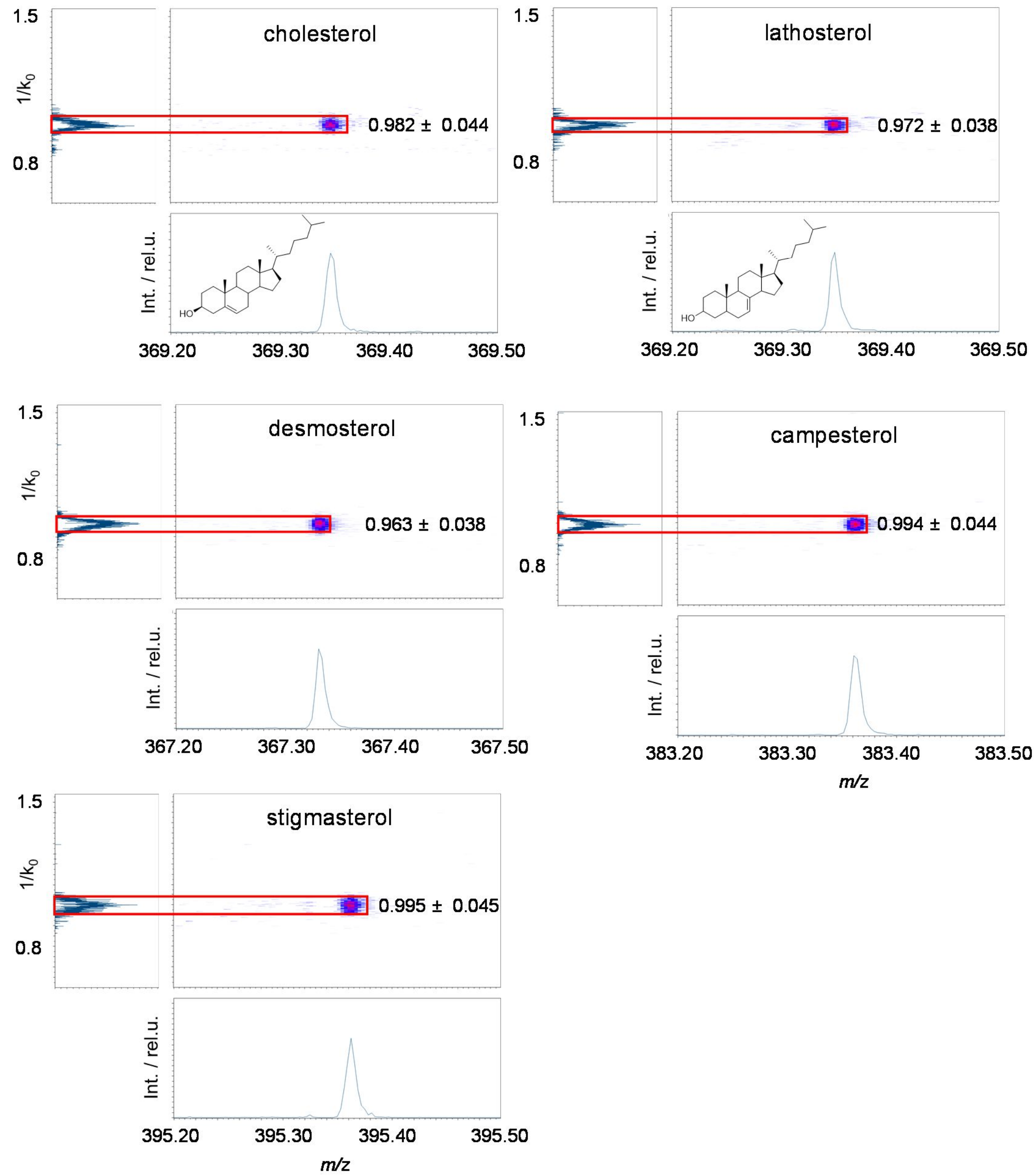

Fig. S4 MALDI-2-TIMS-MS mobilograms of five sterol standards measured with DHAP in dried-droplet preparation. Molecular structures for isobaric cholesterol and lathosterol are isobaric are displayed in the respective spectra (top row).

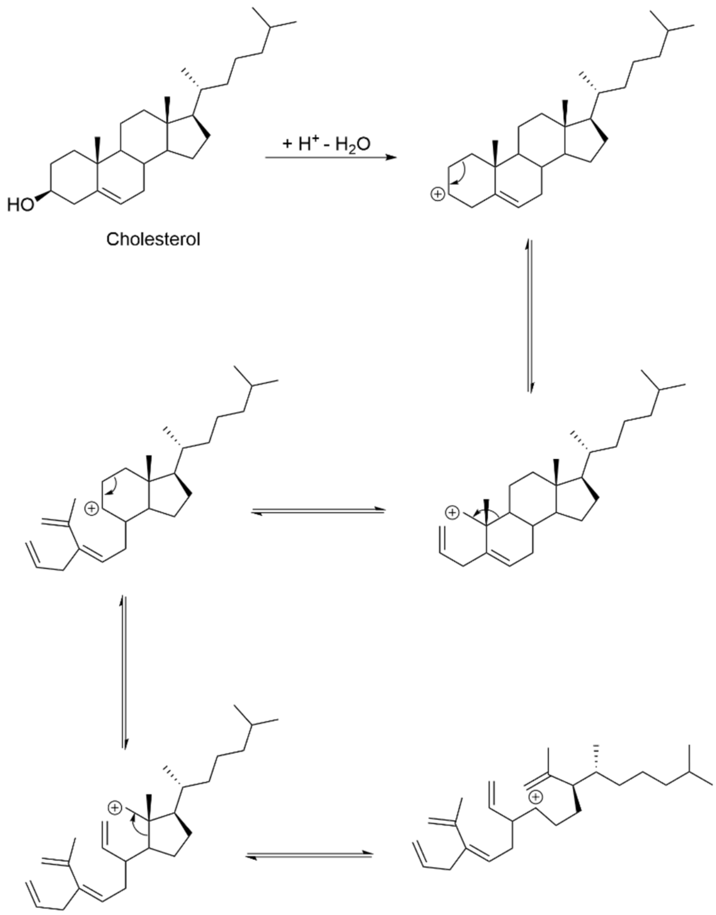

Fig. S5 Putative rearrangement processes of cholesterol that could occur after protonation of the hydroxyl group resulting in several isomeric structures.

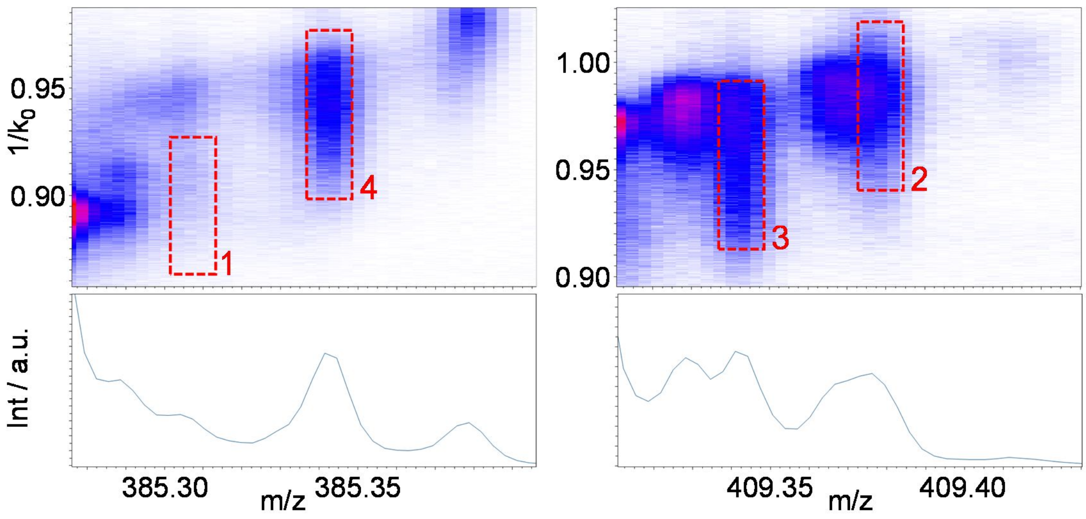

Fig. S6 Examples of mobilogram features associated with sterol ion species graded from one to four. 1: weak signal intensity detected at the predicted m/z-value, no clear feature shape; 2: weak to medium signal intensity detected at the predicted m/z-value, indications of drawn out feature shape, sizable overlap with other feature; 3: medium to strong signal detected at predicted m/z-value, clear drawn out feature shape, weak overlap with other features; 4: medium to strong signal detected at predicted m/z-value, clear drawn out feature shape, no sizeable overlap with other features

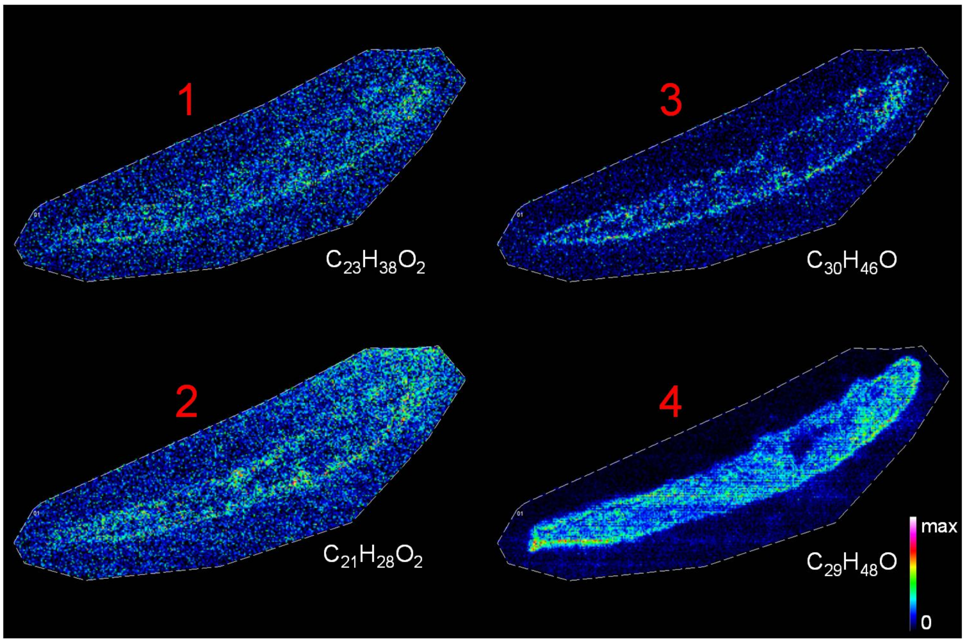

Fig. S7 Examples of signal intensity distributions produced by MALDI-2-TIMS-MSI associated with sterol ion species graded from one to four. 1: Weak signal to noise, outlines of tissue barely discernable; 2: weak to medium signal to noise, outlines of the tissue discernable; 3: medium to high signal to noise, outlines of the tissue clearly discernable, 4: high signal to noise, outlines of the tissue clearly discernable,

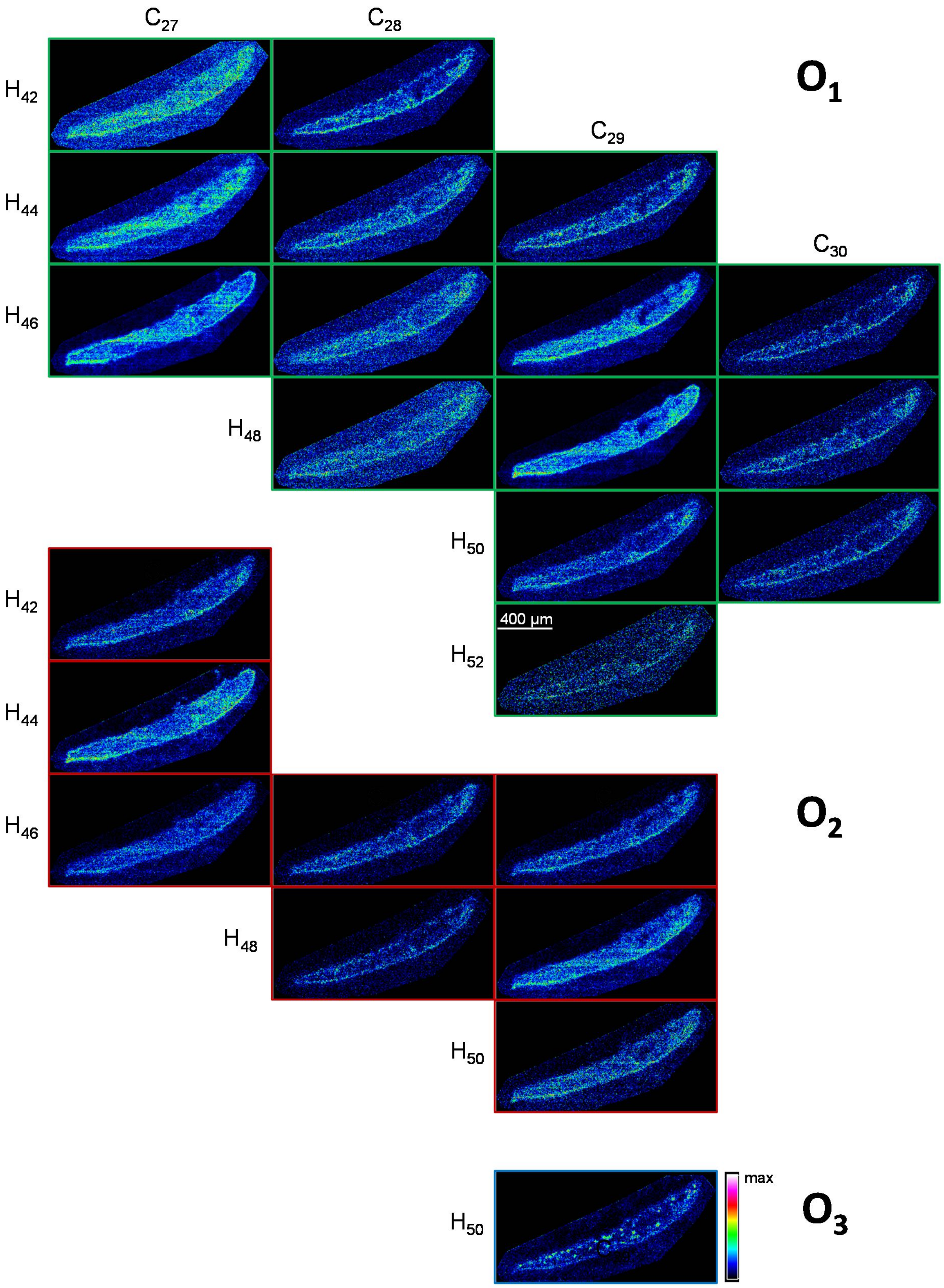

Fig. S8 Exemplary MALDI-2-TIMS-MS images for 24 sterols, sorted by the number of carbon atoms and degree of unsaturation for species containing one, two, or three oxygen. The scale bar is valid for all images displayed.

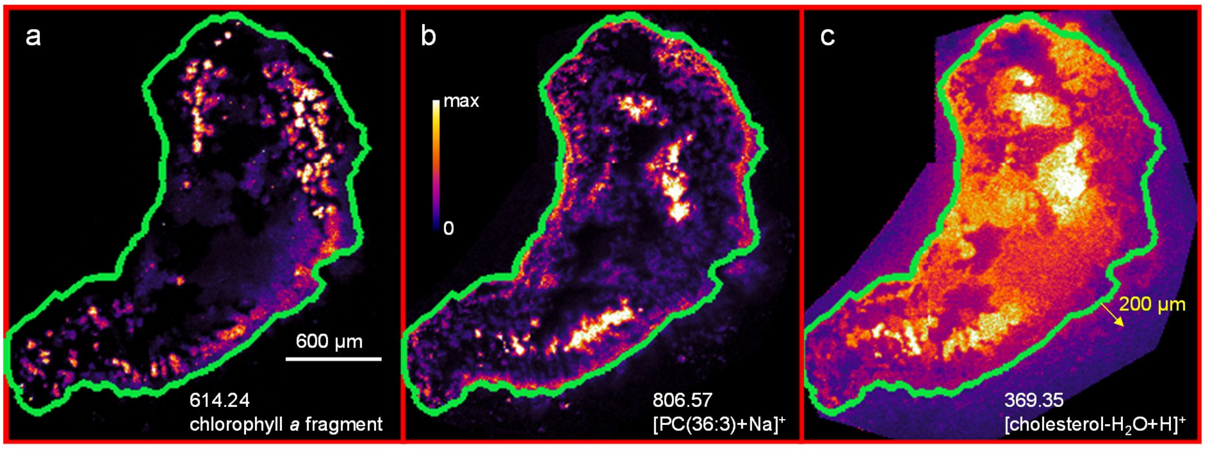

Fig. S9 MALDI-2-MS images of a flatworms that exhibits sizable sterol migration. Images of a chlorophyll a fragment (a) and $[PC(36:3)+Na]^+$ (b) show little delocalization and are indicative of the worm tissue and dinoflagellates, respectively. MALDI-2-MS images cholesterol (c) shows a delocalization of the sterol species up to 200 µm away from the tissue. Green lines indicate the approximate edge of the tissue. Images underwent weak denoising. No normalization was applied.

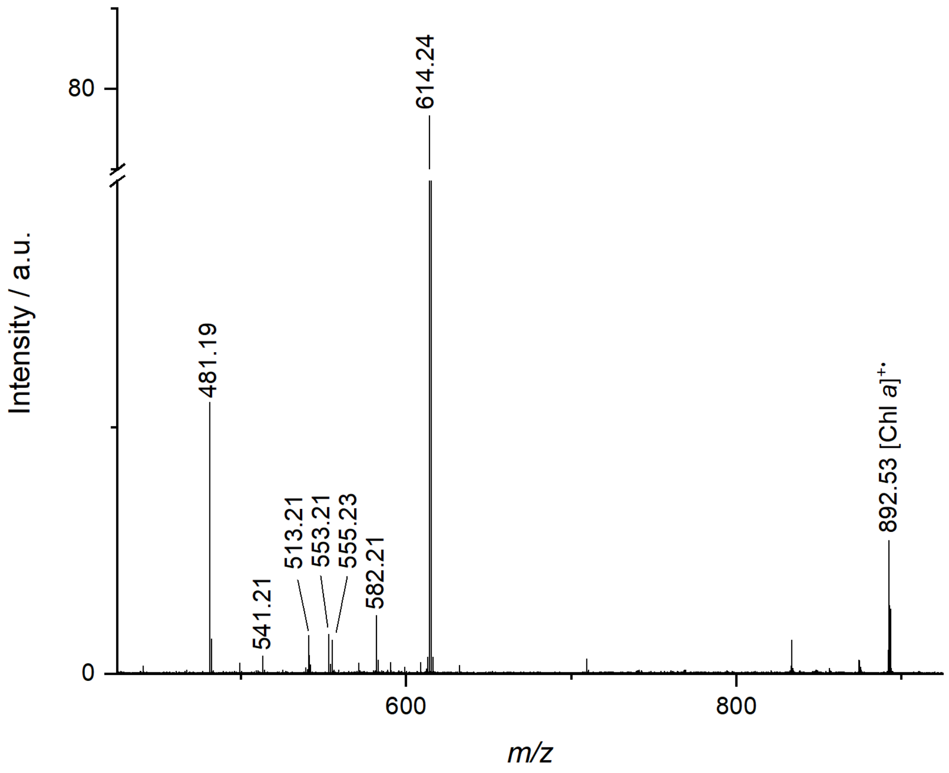

Fig. S10 MSMS-spectrum of a CID-imaging run on a worm section with m/z 892.53 as a precursor mass; fragment at 614 corresponds to chlorophyll *a*, formed upon loss of a neutral phytyl moiety ($C_{20}H_{38}$), and 418 represents the additional loss of a $C_5H_9O_4$-group as described in the literature.(Calvano et al., 2015; Klaproth et al., 2016)

References for the supplementary information

Armoza-Zvuloni, R., Kramarsky-Winter, E., Loya, Y., Schlesinger, A., & Rosenfeld, H. (2014). Trioecy, a Unique Breeding Strategy in the Sea Anemone Aiptasia diaphana and Its Association with Sex Steroids1. *Biology of Reproduction*, *90*(6), 1–8. https://doi.org/10.1095/biolreprod.113.114116

Blunt, J. W., Copp, B. R., Keyzers, R. A., Munro, M. H. G., & Prinsep, M. R. (2013). Marine natural products. *Nat. Prod. Rep.*, *30*(2), 237–323. https://doi.org/10.1039/C2NP20112G

Calderón, G. J., Castellanos, L., Duque, C., Echigo, S., Hara, N., & Fujimoto, Y. (2004). Ophirasterol, a new C31 sterol from the marine sponge Topsentia ophiraphidites. *Steroids*, *69*(2), 93–100. https://doi.org/10.1016/j.steroids.2003.11.001

Calvano, C. D., Ventura, G., Cataldi, T. R. I., & Palmisano, F. (2015). Improvement of chlorophyll identification in foodstuffs by MALDI ToF/ToF mass spectrometry using 1,5-diaminonaphthalene electron transfer secondary reaction matrix. *Analytical and Bioanalytical Chemistry*, *407*(21), 6369–6379. https://doi.org/10.1007/s00216-015-8728-9

Kerr, R. G., & Baker, B. J. (1991). Marine sterols. *Natural Product Reports*, *8*(5), 465. https://doi.org/10.1039/np9910800465

Klaproth, A., Najdanova, M., Minceva, M., Sicker, D., Siehl, H.-U., Zeller, K.-P., & Berger, S. (2016). Chlorophyll. *Chemie in Unserer Zeit*, *50*(4), 260–274. https://doi.org/10.1002/ciuz.201600751

Ohnishi, T., Yokota, T., & Mizutani, M. (2009). Insights into the function and evolution of P450s in plant steroid metabolism. *Phytochemistry*, *70*(17–18), 1918–1929. https://doi.org/10.1016/j.phytochem.2009.09.015

Revel, J., Massi, L., Mehiri, M., Boutoute, M., Mayzaud, P., Capron, L., & Sabourault, C. (2016). Differential distribution of lipids in epidermis, gastrodermis and hosted Symbiodinium in the sea anemone Anemonia viridis. *Comparative Biochemistry and Physiology Part A: Molecular & Integrative Physiology*, *191*, 140–151. https://doi.org/10.1016/j.cbpa.2015.10.017

Sjövall, J., & Danielsson, H. (1985). *Sterols and Bile Acids (New Comprehensive Biochemistry; V. 12)*. Elsevier Science Limited.
